# Physical activity is a potential measure of physical resilience in older adults receiving hemodialysis

**DOI:** 10.3389/fneph.2022.1032468

**Published:** 2023-01-06

**Authors:** Anika Lucas, Jeanette Rutledge, Richard Sloane, Katherine Hall, Ciara Green, Carl Pieper, Cathleen Colón-Emeric, Rasheeda Hall

**Affiliations:** ^1^ Durham Veterans Affairs Healthcare System, Renal Section, Durham, NC, United States; ^2^ Department of Medicine, Division of Nephrology, Duke University, Durham, NC, United States; ^3^ Center for the Study of Aging and Human Development, Duke University, Durham, NC, United States; ^4^ Durham Veterans Affairs Healthcare System, Geriatric Research Education and Clinical Center, Durham, NC, United States; ^5^ Department of Medicine, Division of Geriatrics, Duke University, Durham, NC, United States

**Keywords:** physical activity, physical function, older adults, hemodialysis, resilience

## Abstract

**Background:**

Physical resilience, or the ability to recover after a physical stressor, declines with aging. Efforts to preserve physical resilience in the older dialysis population are critically needed; however, validated, patient-centered measures that are sensitive to change are also needed. Our objective was to assess accelerometer-derived step count variability, or a measure of intra-individual variation in physical activity, as a potential measure of physical resilience among older adults receiving hemodialysis.

**Methods:**

Community-dwelling ambulatory older adults receiving in-center hemodialysis were prospectively enrolled. Participants wore wrist accelerometers during daytime hours on both dialysis and non-dialysis days up to 14 days, and the feasibility of accelerometer use was assessed from wear time. We used accelerometer data to compute step counts in 4-hour blocks and step count variability. Physical function was assessed with the Short Physical Performance Battery (SPPB which includes gait speed test), grip strength, activities of daily living (ADLs) instruments, and life space mobility. We assessed interval fatigue (subjective rating from 0 to 10) on dialysis and non-dialysis days and self-reported recovery time. We assessed the correlations of step count variability with measures of physical function and step count and interval fatigue.

**Results:**

Of 37 enrolled participants, 29 had sufficient accelerometer data for analyses. Among the 29 participants, mean (SD) age was 70.6(4.8) years, and 55% (n=16) were male and 72% (n=21) were Black race. Participants were largely sedentary with median (Q1-Q3) self-reported total kilocalories per week of 200 (36–552). Step count variability was positively correlated with measures of physical function: SPPB (r=0.50, p<0.05), gait speed (r=0.59, p<0.05), handgrip strength (r=0.71, p<0.05), Instrumental ADLs (r=0.44, p<0.05) and life space mobility (r=0.54, p<0.05).There was a weak inverse correlation between post-dialysis step counts (4-hour blocks after a dialysis session) and post-dialysis interval fatigue [r=-0.19 (n=102, p=0.06).

**Conclusions:**

Physical activity assessment *via* accelerometer is feasible for older adults receiving hemodialysis. Step count variability correlated with physical function, so it may be a novel measure of physical resilience. Further studies are needed to validate this measure.

## Introduction

Among older adults receiving hemodialysis, nearly 40% experience a decline in physical function in a given year (1). The remaining 60% maintain or improve their physical function. This heterogeneity is likely in part explained by variations in physical resilience (2). Physical resilience encompasses the ability to maintain or recover functioning after a physical stressor (2). Studies of physical resilience in older dialysis patients may elucidate clues for maintaining physical function and quality of life (3, 4). However, there is no unifying understanding of physical resilience or validated measures of physical resilience in older adults receiving hemodialysis.

Physical resilience for a hemodialysis patient can manifest as an individual’s ability to recover after the hemodynamic and pro-inflammatory stress imposed by each hemodialysis session (5). We hypothesized that physical resilience, or degree of recovery, would be reflected by how activity patterns recover to baseline following a hemodialysis session. Accelerometry has been used to assess activity patterns through step counts in hemodialysis patients in prior studies (6–8). Therefore, step count variability, or a measure of intra-individual variation in step counts in discrete time intervals, could reflect recovery after hemodialysis. For example, a patient with higher step count variability would have wider shifts in physical activity across intervals because they spent more time in a day active than resting. As in prior studies of dynamical systems (e.g. heart rate variability, inflammatory response) (9, 10), higher step count variability reflects higher physiological complexity, or an individual’s ability to demonstrate physiological adaptations after a stressor, which is considered a hallmark of successful aging (11–13). Understanding if step count variability can provide information suggestive of physical resilience is valuable for development of resilience measures for older adults receiving hemodialysis.

The primary objective of this study was to determine the role of physical activity assessment *via* accelerometer in physical resilience. To achieve this objective, we conducted a prospective cohort study in older adults receiving hemodialysis to assess the feasibility of accelerometer use and the relationship between step count variability and physical function. Because accelerometer use can be impractical, we also explored additional measures (e.g., serial measures of fatigue, self-reported recovery time) and their relationship with physical activity to identify potential alternative measures.

## Materials and methods

This was a prospective, longitudinal feasibility study that aimed to evaluate physical activity as a potential measure of physical resilience in older patients receiving in-center hemodialysis. We recruited a convenience sample of 37 older adults (≥ 65 years) receiving hemodialysis sessions from 2017 to 2018. Exclusion criteria included individuals who were not ambulatory, those who did not exhibit independence in all ADLs (e.g., long-term care residents), those with advanced dementia, non-English speaking individuals, and those in hospice. All subject screening, recruitment, and consent occurred at outpatient dialysis clinics within 32 miles of Duke University Hospital. This protocol was approved by the Duke IRB (Pro 00075802), and all patients provided written informed consent.

### Physical activity

Participants underwent home physical activity monitoring for 14 consecutive days using a wrist-mounted Actigraph accelerometer (models GT3X and GT3X+; Pensacola, FL). The Actigraph triaxial accelerometer has been widely validated as a reliable instrument to estimate daily physical activity (14).

Activity monitors and instructions were given to participants during an initial study visit on a typical dialysis session day. Study personnel demonstrated wrist placement and explained the accelerometer to the participants using a standardized script. Participants wore the device during day-time hours, except at times when there was a possibility of the device becoming wet. The accelerometer was worn on non-access wrist (a wrist that does not have a functional arteriovenous access for dialysis) attached to a wrist band.

### Alternative measures of physical resilience

We postulated that alternative measures of physical resilience would include interval fatigue and self-reported recovery time (15). As in other studies evaluating fatigue variability in the dialysis population (16, 17), interval fatigue was assessed during the period of home physical activity monitoring. Study personnel called and/or texted participants at three regular intervals throughout each weekday (e.g., 10AM, 2PM, and 6PM) to ask participants to report their fatigue score at that moment on a numeric rating scale (0–10). A score of 0 indicated “no fatigue” and a score of 10 “fatigue as bad as you can imagine.” (18) We also assessed self-reported recovery time by asking the following validated question: “How long does it take for you to recover from a dialysis session?” (19)

### Additional measures

To obtain a baseline assessment of physical function, we administered the following measures in the dialysis unit (before dialysis on a mid-week dialysis day): Short Physical Performance Battery (SPPB), [SPPB includes assessments of gait speed, time to complete 5 chair stands, and balance. All tests in the SPPB are scored 0-4, for a total score of 0-12, with higher scores indicating better function], handgrip strength (as measured using a dynamometer), Lawton (20) and Katz (21) ADLs, and Life-Space Mobility (22). We also collected demographics, comorbidities using the Charlson Comorbidity Index, and length of time on dialysis. Participants were also asked to complete the Low Physical Activity Questionnaire, an instrument that converts survey responses about physical activity into total kilocalories(kcal)/week (23).

### Statistical analyses

**Figure 1:**
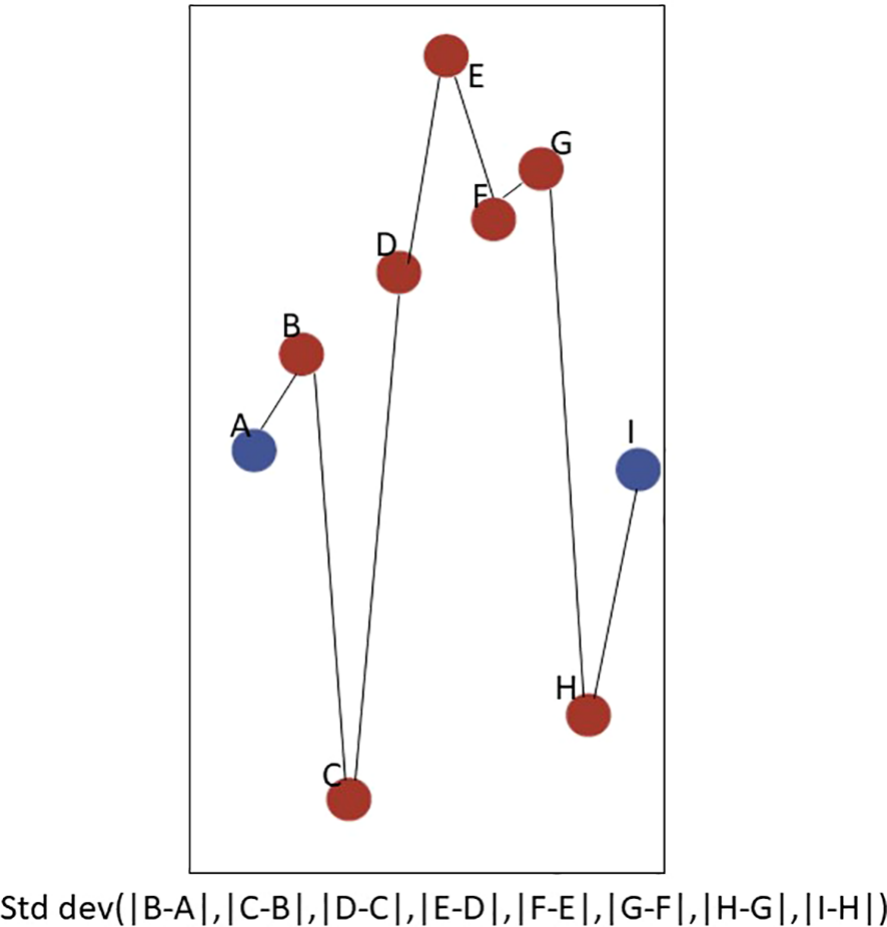
Schematic of Step Count Variability Measure Using a Participant’s Data. Here is an example of a subset of 4-hour blocks (blue indicates dialysis block, and red indicates non-dialysis block) between two consecutive dialysis sessions for one participant. Step count differences between each 4-hour block were calculated for each participant. Then, the step count variability was calculated as the standard deviation of the series of step count differences for each participant.

Using ActiLife v6.12 data processing software, accelerometer physical activity data was processed into step counts (24). Only individuals who had ≥ 10 hours of valid wear time on 3 or more days were retained for analyses (25). To assess changes in step counts over an interdialytic period, participants had to have at least 3 days of data that included both dialysis and non-dialysis days. Step counts were summed in 4-hour (4-hr) blocks. With knowledge of each participant’s dialysis schedule, each participant’s hemodialysis session was represented in one 4-hr block (see blue dots in **Figure 1**). Then, all other 4-hr blocks, or non-dialysis blocks, were assigned relative to the participant’s hemodialysis 4-hr block. In an approach similar to heart rate variability (9, 26), step count variability was estimated from the difference in step counts from one 4-hr block to another (each dot in **Figure 1**). Each participant had a series of step count differences determined over all of their 4-hr blocks, and step count variability for each participant was calculated as the standard deviation of the absolute value of all of the step count differences (formula in **Figure 1**). Similar to heart rate variability, higher step count variability reflects higher physiological complexity which is essential for recovery from a stressor (9).

Analyses were conducted using data from participants with sufficient step count data. Summary statistics are presented as mean ± SD or median (Q1-Q3) for continuous data and presented as proportions for categorical data. To assess feasibility of accelerometer use, we calculated the average time participants wore it out of the expected 14 days. We assessed feasibility of interval fatigue assessment from the proportion of participants with any missing interval fatigue data. Assumptions allowing the use of parametric analysis were largely met in order to perform a Pearson correlation to assess the relationship between step counts and step count variability (measured in 4-hr blocks). We visualized the relationship between step count variability by tertiles and step count during hemodialysis and post-dialysis 4-hr blocks over three consecutive dialysis days. To assess step count variability as a measure of physical resilience, we assessed Pearson correlations between step count variability and physical function (SPPB total score, gait speed, hand grip strength, ADLS, and life space mobility). These correlations were conducted using both Pearson and Spearman correlations, with similar results for both. For ease of interpretation we are presenting the Pearson correlation results.

We assessed correlations of physical activity and alternative measures of physical resilience including fatigue. Because fatigue was not normally distributed, Spearman’s correlation was used to assess the association between step count (measured in 4-hr blocks) and interval fatigue for both post-dialysis 4-hr blocks and for all other 4-hr blocks. We assessed the correlation between step count variability and self-reported recovery time and total kcal/week (derived from the Low Physical Activity Questionnaire). All statistical analyses were performed using SAS (version 9.4, SAS Inc., Cary NC).

## Results

### Cohort characteristics

**Figure 2:**
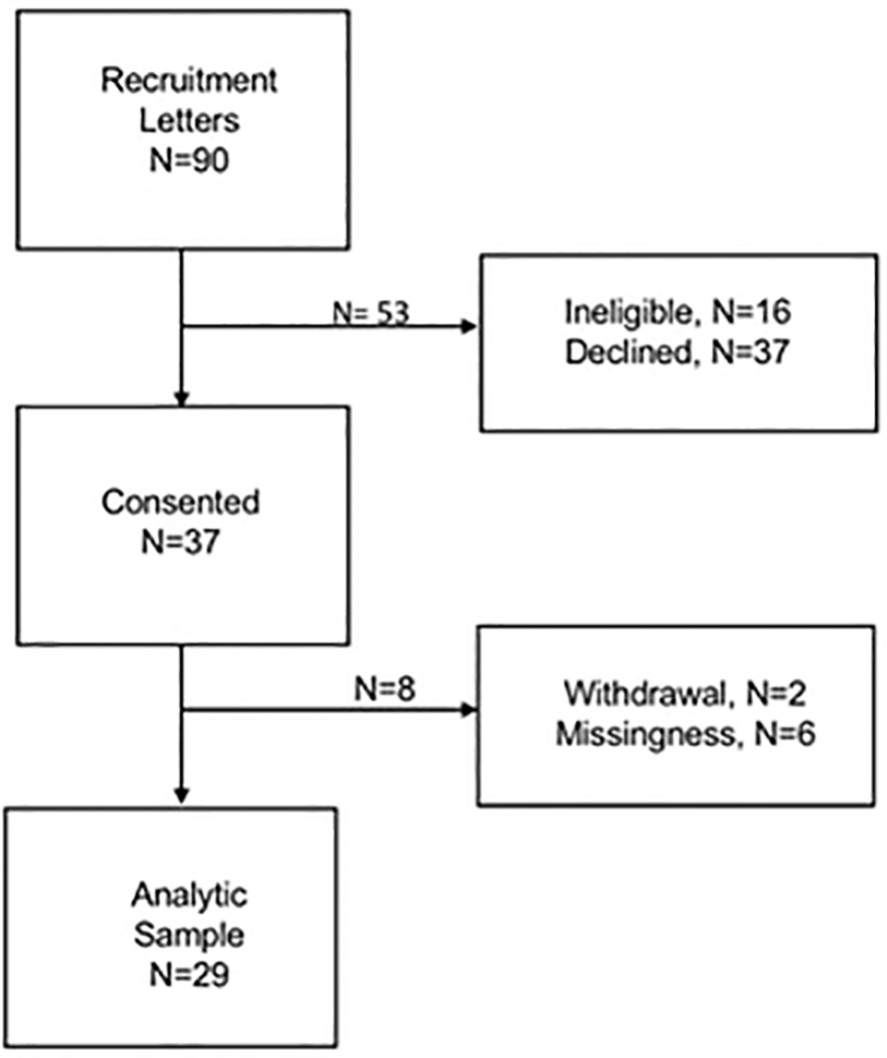
Flowchart of study participants .

Of 37 participants, 8 were excluded from analyses for insufficient data (wearing accelerometer < 3 days, or only on dialysis days (n=6), or study withdrawal due to illness (n=2)) (**Figure 2**). Among the 29 participants in the analytic cohort, mean (SD) age was 70.6 (4.8) years, 55% (n=16) were men, and 72% (n=21) were Black race (**Table 1**). Participants had mean (SD) SPPB 6.3 (3.2), mean (SD) life-space mobility score 21.8 (8.4) and self-reported caloric expenditure was median (Q1-Q3) 200 (36-552) total kcal/week. While participants generally had preserved basic ADLs, instrumental ADLs, and handgrip strength (28); there was heterogeneity in self-reported recovery time [median (Q1-Q3): 2 (0, 24) hours].

**Table 1:** Baseline characteristics of participants.

Patient Characteristics	Mean (SD), Median (Q1-Q3) or N (%)		
	Lowest Tertile (N=10)	Middle Tertile (N=9)	Highest Tertile (N=10)
Age	71.2 (5.5)	70.3 (2.7)	70.3 (5.9)
Male sex	4 (40%)	4 (44%)	8 (80%)
Black race	6 (60%)	8 (89%)	7 (70%)
Time on Dialysis (years) ^a^	3.8 (0.6-4.9)	2.6 (1.6-4.9)	2.6 (1.5-6.2)
Charlson Index	4.8 (1.5)	4.8 (1.8)	4.0 (1.8)
Hemoglobin	10.2 (1.6)	10.8 (0.9)	10.7 (0.7)
Kt/V	1.6 (0.4)	1.7 (0.1)	1.5 (0.1)
Short Physical Performance Battery (SPPB)^b^	4.4 (2.1)	5.7 (3.5)	8.5 (2.5)
Gait Speed	0.5 (0.2)	0.7 (0.3)	0.9 (0.2)
Handgrip Strength (kilograms)	44.2 (13.7)	56.3 (13.3)	71.3 (10.4)
Basic Activities of Daily Living (ADL) Score	5.6 (1.0)	5.9 (0.4)	6 (0)
Instrumental Activities of Daily Living Score	4.9 (2.1)	7.9 (0.4)	7.3 (1.3)
Life Space Mobility Score^c^	18.1 (6.8)	19.9 (8.0)	27.2 (8.1)
Self-reported Recovery Time (SRRT) (minutes) **^a,d^**	480 (120-720)	60 (5-180)	120 (25-450)
Total kilocalories per week (kcal/week) **^a,e^**	18 (0-270)	300 (110-560)	407 (165-670)
Step Count Variability	97.7 (19.2)	151.6 (8.2)	262 (64.1)

a Median (Q1-Q3); N=29; SPPB range 0-12; max Basic ADL score 6; max Instrumental ADL score 8.

b SPPB ^<^10 indicates higher odds of future loss of ability to walk 400 m (25).

c Life space mobility score <30 indicates someone who needs help going beyond their own yard (18).

d Max SRRT 1440 min/day.

e This metric reflects energy expenditure from physical activity derived from the Low Physical Activity Questionnaire (19). Low physical activity is <383 kcal/wk. for men or 270 kcal/week for women (27).

### Feasibility

On average, participants wore accelerometer on their wrist 14.9 (1.1) hours a day over 12.9 (1.9) days. Although one of the participants excluded from analyses noted the device “bothered” them, none of the participants included in our analyses reported side effects. The majority of participants (25 of 29) had some missing interval fatigue data.

### Step counts and their correlation with step count variability

**Figure 3:**
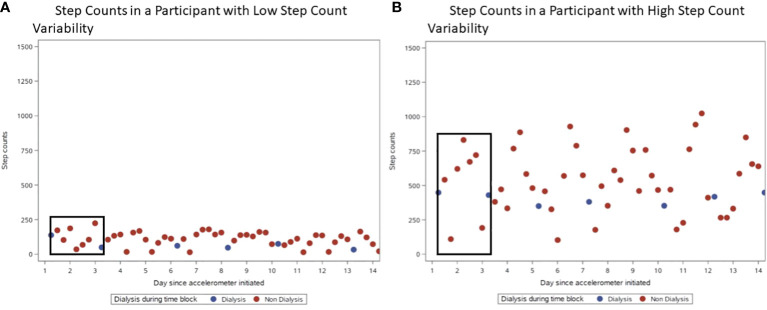
Examples of Physical Activity Data at 4-hour blocks. For both plots from actual study participants, the blue dots indicate dialysis 4-hour blocks, and the red dots indicate non-dialysis 4-hour blocks. Black boxes demonstrate a set of 4-hour blocks between two consecutive dialysis sessions. **(A)** Step Counts in a Participant with Low Step Count Variability. **(B)** Step Counts in a Participant with High Step Count Variability.

**Figure 4:**
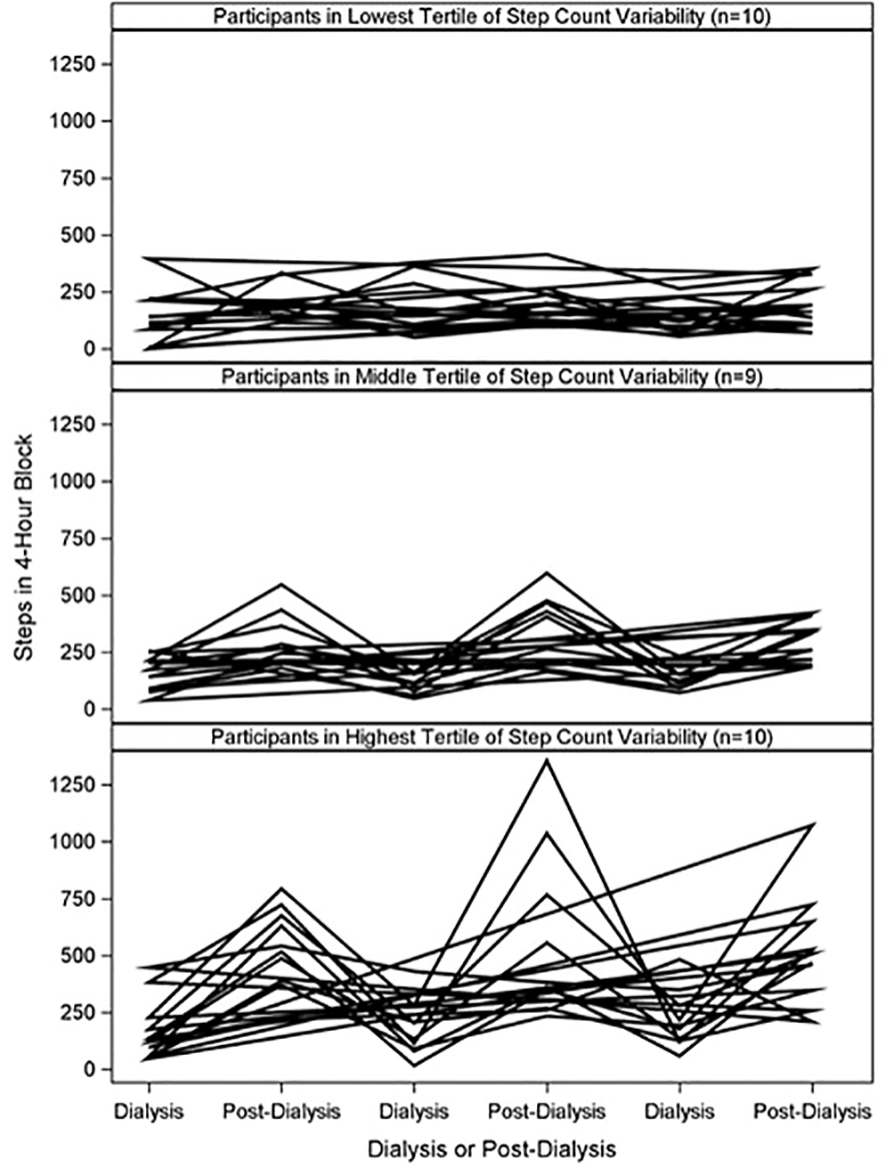
Individual Step Counts at Dialysis and Post-Dialysis Blocks by Tertile of Step Count Variability. Participants were grouped into tertiles of step count variability. Plot shows step counts at dialysis and post-dialysis 4-hour blocks from three distinct dialysis days.

**Figure 3** shows example patterns of step counts at 4-hr blocks:1) a participant with similar step counts during dialysis 4-hr blocks and other 4-hour blocks (**Figure 3A**) and 2) a participant with higher step counts at 4-hr blocks outside of dialysis in most blocks (**Figure 3B**). There was a strong, positive correlation between step count and step count variability (Pearson’s r=0.89, p<0.0001). The mean step count variability was 140.0 ± 67.3 steps, and participants within highest tertile of step count variability had higher step counts in post-dialysis 4-hr blocks compared to those in lower tertiles (**Figure 4**).

### Step count variability and its correlation with measures of physical function

Step count variability had a positive Pearson correlation with physical function measures: SPPB, gait speed, handgrip strength, ADLs and life space mobility (**Table 2**). Highest correlations were with objective measures of physical function: gait speed (Pearson’s r=0.59, p-value=0.001) and handgrip strength (Pearson’s r=0.71, p-value<0.0001).

**Table 2:** Pearson Correlations between Step Count Variability and Measures of Physical Function and/or Resilience ^a^.

Measures of Physical Function and/or Resilience	Correlation r	p-value
Short Physical Performance Battery	0.5	0.006
Gait speed	0.59	0.001
Handgrip strength	0.71	<0.0001
Instrumental ADLs	0.44	0.02
Basic ADLs	0.24	0.23
Total kcal/week	0.48	0.009
Self-Reported Recovery Time	-0.17	0.38

^a.^Total kcal/week derived from the Low Physical Activity Questionnaire (LoPAQ).

### Step count/step count variability and correlation with alternative measures of physical resilience

Median (Q1-Q3) interval fatigue after hemodialysis was slightly higher than fatigue reported on non-dialysis days [4(2-6) vs. 3(1-5)]. There was a weak, inverse Spearman correlation between step counts and interval fatigue in the 4-hr block after hemodialysis [r=-0.19 (n=102, p=0.06)], and, for step counts and interval fatigue at all other 4-hr blocks [r=-0.17 (n=210, p=0.01)].

Step count variability had a positive, moderate Pearson correlation with total kcal/week (from Low Physical activity Questionnaire) but weak, inverse Pearson correlation with self-reported recovery time (**Table 2**).

## Discussion

In this study, we found it is feasible to measure physical activity *via* accelerometer in an older dialysis cohort. Step count variability, a measure that reflects how activity patterns return to baseline after hemodialysis, had moderate to high correlations with physical function measures: hand grip strength, gait speed, life space mobility, SPPB, and ADLs. These findings suggest that step count variability could be a measure of physical resilience in older adults receiving dialysis. Additional work is needed to confirm that step count variability is sensitive to change with resilience-promoting interventions and therefore reasonable target for efforts to maintain or improve physical resilience in this population.

This study uniquely identifies step count variability as a potential measure of physical resilience based on the correlations between step count variability and physical function measures in older adults receiving dialysis. Prior studies in both a general older adult cohort and a dialysis exercise intervention cohort also demonstrate a positive correlation between physical activity (either exercise intensity or accelerometer) and physical function measured by physical performance measures or questionnaire (27, 29). In a similar study design, Majchrzak et al. found physical activity and physical function to be positively correlated, however they found physical activity to be similar on both dialysis and non-dialysis days (6). Our study provides novel information by exploring recovery of physical activity after dialysis through step count variability, instead of step counts alone. Using step count variability, we found that the extent of physical activity within 4-hr blocks does vary among older adults when they are not receiving hemodialysis and can indicate better physical function even in older participants who on average had low physical function based on SPPB and life-space mobility scores (22, 30).

Prior evidence suggests self-reported fatigue and recovery time would be inversely related to physical activity (19) (15, 31). However, our study showed self-reported fatigue and recovery time instruments, while less resource-intensive than accelerometer use, had only weak inverse correlations with physical activity. These findings could be partially explained by the fact that interval fatigue assessment could not be reliably obtained in this population and the small sample size. The weak correlations may also partially be explained by their inherent subjectivity. Some patients receiving dialysis may rate fatigue or recovery time that does not correspond with their activity level for a myriad of reasons to stay active despite fatigue (e.g., caregiving responsibilities, employment) (32). Also, fatigue and recovery time may be interpreted differently from person to person due to culture or other individual attributes (33). Despite the findings from this pilot study, fatigue and post-dialysis recovery are important patient-centered outcomes identified by the hemodialysis population (34, 35). Future work is needed to evaluate interval fatigue and recovery time as potential measures of physical resilience. For now, clinicians and researchers should consider fatigue and low physical activity as separate, although related issues, in clinical evaluation and outcome measures.

While most physical activity studies in the dialysis population have been conducted in patients 1-2 decades younger than those in this cohort (31, 32), our study diminishes concern about the feasibility of accelerometer use in older adults receiving dialysis. There may be concern that older adults may not be able to follow instructions and adhere to the study protocol; however, the majority (>80%) followed instructions, wearing the device for nearly 15 hours a day, and had eligible data for analyses. Another concern would be that older adults receiving dialysis are less active (demonstrated by the low self-reported total kcal/week by participants (36)) than younger patients, so the data may not be robust enough to identify differences within a sedentary group. However, we found clear correlations with physical function measures. Given these early findings, additional studies are needed to validate this measure against other clinical outcomes thought to reflect physical resilience (e.g., recovery after elective surgery).

Then, subsequent applications of step count variability could involve use as an outcome measure in studies designed to improve physical resilience. Such work would be useful as often physical activity improvement may precede other measurable improvements (e.g., physical function or quality of life). Additionally, step count variability could be applied in identification of high risk patients as low physical resilience phenotypes have been associated with adverse outcomes in older adults receiving hemodialysis (37).

This study’s strength lies in its unique cohort of older Black adults receiving hemodialysis (average age 71) who are not routinely included in research studies. Our study had several limitations. First, our measure of interval fatigue on a scale ranging from 0 to 10, is not validated, subject to measurement error, and interval fatigue data was missing in most participants. As a result, we were not able to explore additional analyses of fatigue as a measure of physical resilience or evaluate correlation with physical function. Additional studies for measuring interval fatigue should explore formal psychometric evaluation to create a more valid measure and approach for data collection. The existing Standardized Outcomes in Nephrology-Hemodialysis fatigue instrument could be an alternative measure for interval fatigue after additional studies confirm the instrument’s responsiveness to change and its validation in diverse populations (38). Then, further research could more adequately evaluate the relationships of fatigue with physical function and physical resilience. Second, some participants were excluded for not wearing accelerometer as directed. Future use of accelerometers, especially in an older study population, should require additional reminders and/or staff support to improve study protocol adherence. Third, physical activity may have been influenced by seasonal differences, however we still observed that step count variability was correlated with physical function. Last, the small sample size makes our findings susceptible to Type 1 and Type 2 error, such that these findings should be evaluated in a larger sample to confirm validity.

In conclusion, we identified step count variability as a potential measure of physical resilience for older adults receiving dialysis. This study provides a significant contribution for measure selection in future studies of physical resilience in the dialysis population. Such studies are needed to establish evidence for maintenance or improvement of physical resilience in older adults receiving hemodialysis.

## Data availability statement

The raw data supporting the conclusions of this article will be made available by the authors, without undue reservation.

## Ethics statement

The studies involving human participants were reviewed and approved by Duke University IRB. The patients/participants provided their written informed consent to participate in this study.

## Author contributions

RH, KH, RS, CP, CC-E contributed to conception and study design. JR and RH collected data. CG, KH, RH organized the data. RS performed statistical analyses. AL and RH wrote the first draft of the manuscript. All authors contributed to manuscript revision, read, and approved the submitted version.

## References

[1] Hall RK Luciano A Pendergast JF Colon-Emeric CS. Self-reported physical function decline and mortality in older adults receiving hemodialysis. Kidney Med (2019) 1(5):288–95. doi: 10.1016/j.xkme.2019.08.001 PMC7380442 32734209

[2] Whitson HE Duan-Porter W Schmader KE Morey MC Cohen HJ Colón-Emeric CS. Physical resilience in older adults: Systematic review and development of an emerging construct. J Gerontol A Biol Sci Med Sci (2016) 71(4):489–95. doi: 10.1093/gerona/glv202 PMC5014191 26718984

[3] Colón-Emeric C Whitson HE Pieper CF Sloane R Orwig D Huffman KM. Resiliency groups following hip fracture in older adults. J Am Geriatr Soc (2019) 67(12):2519–27. doi: 10.1111/jgs.16152 PMC6898748 31469411

[4] Duan-Porter W Cohen HJ Demark-Wahnefried W Sloane R Pendergast JF Snyder DC. Physical resilience of older cancer survivors: An emerging concept. J Geriatr Oncol (2016) 7(6):471–8. doi: 10.1016/j.jgo.2016.07.009 PMC5159214 27478133

[5] Jofre R Rodriguez-Benitez P Lopez-Gomez JM Perez-Garcia R. Inflammatory syndrome in patients on hemodialysis. J Am Soc Nephrol (2006) 17(12 Suppl 3):S274–80. doi: 10.1681/ASN.2006080926 17130274

[6] Majchrzak KM Pupim LB Chen K Martin CJ Gaffney S Greene JH. Physical activity patterns in chronic hemodialysis patients: comparison of dialysis and nondialysis days. J Ren Nutr (2005) 15(2):217–24. doi: 10.1053/j.jrn.2004.08.002 15827895

[7] Gomes EP Reboredo MM Carvalho EV Teixeira DR Carvalho LF Filho GF. Physical activity in hemodialysis patients measured by triaxial accelerometer. BioMed Res Int (2015) 2015:645645. doi: 10.1155/2015/645645 26090432 PMC4458275

[8] Shiota K Hashimoto T. Promotion and support of physical activity in elderly patients on hemodialysis: a case study. J Phys Ther Sci (2016) 28(4):1378–83. doi: 10.1589/jpts.28.1378 PMC4868247 27190487

[9] Chaves PH Varadhan R Lipsitz LA Stein PK Windham BG Tian J. Physiological complexity underlying heart rate dynamics and frailty status in community-dwelling older women. J Am Geriatr Soc (2008) 56(9):1698–703. doi: 10.1111/j.1532-5415.2008.01858.x PMC2848445 19166446

[10] Whitson HE Crabtree D Pieper CF Ha C Au S Berger M. A template for physical resilience research in older adults: Methods of the PRIME-KNEE study. J Am Geriatr Soc (2021) 69(11):3232–41. doi: 10.1111/jgs.17384 PMC8595699 34325481

[11] Merchant RA Aprahamian I Woo J Vellas B Morley JE. Editorial: Resilience and successful aging. J Nutr Health Aging (2022) 26(7):652–6. doi: 10.1007/s12603-022-1818-4 PMC9209635 35842754

[12] Varadhan R Walston JD Bandeen-Roche K. Can a link be found between physical resilience and frailty in older adults by studying dynamical systems? J Am Geriatr Soc (2018) 66(8):1455–8. doi: 10.1111/jgs.15409 PMC6133741 29727469

[13] Whitson HE Cohen HJ Schmader KE Morey MC Kuchel G Colon-Emeric CS. Physical resilience: Not simply the opposite of frailty. J Am Geriatr Soc (2018) 66(8):1459–61. doi: 10.1111/jgs.15233 PMC6157007 29577234

[14] Jarrett H Fitzgerald L Routen AC. Interinstrument reliability of the ActiGraph GT3X+ ambulatory activity monitor during free-living conditions in adults. J Phys Act Health (2015) 12(3):382–7. doi: 10.1123/jpah.2013-0070 24828685

[15] Jhamb M Weisbord SD Steel JL Unruh M. Fatigue in patients receiving maintenance dialysis: a review of definitions, measures, and contributing factors. Am J Kidney Dis (2008) 52(2):353–65. doi: 10.1053/j.ajkd.2008.05.005 PMC2582327 18572290

[16] Brys ADH Lenaert B Van Heugten CM Gambaro G Bossola M. Exploring the diurnal course of fatigue in patients on hemodialysis treatment and its relation with depressive symptoms and classical conditioning. J Pain Symp Manage (2019) 57(5):890–8.e4. doi: 10.1016/j.jpainsymman.2019.02.010 30776536

[17] Abdel-Kader K Jhamb M Mandich LA Yabes J Keene RM Beach S. Ecological momentary assessment of fatigue, sleepiness, and exhaustion in ESKD. BMC Nephrol (2014) 15:29. doi: 10.1186/1471-2369-15-29 24502751 PMC3927224

[18] Murphy SL Alexander NB Levoska M Smith DM. Relationship between fatigue and subsequent physical activity among older adults with symptomatic osteoarthritis. Arthritis Care Res (2013) 65(10):1617–24. doi: 10.1002/acr.22030 PMC3787954 23592576

[19] Lindsay RM Heidenheim PA Nesrallah G Garg AX Suri R. Daily hemodialysis study group London health sciences c. minutes to recovery after a hemodialysis session: a simple health-related quality of life question that is reliable, valid, and sensitive to change. Clin J Am Soc Nephrol (2006) 1(5):952–9. doi: 10.2215/CJN.00040106 17699312

[20] Lawton MP Brody EM. Assessment of older people: self-maintaining and instrumental activities of daily living. Gerontologist (1969) 9(3):179–86. doi: 10.1093/geront/9.3_Part_1.179 5349366

[21] Katz S Ford AB Moskowitz RW Jackson BA Jaffe MW. Studies of illness in the aged. the index of adl: a standardized measure of biological and psychosocial function. Jama (1963) 185:914–9. doi: 10.1001/jama.1963.03060120024016 14044222

[22] Peel C Baker PS Roth DL Brown CJ Bodner EV Allman RM. Assessing mobility in older adults: The UAB study of aging life-space assessment. Phys Ther (2005) 85(10):1008–19. doi: 10.1093/ptj/85.10.1008 16180950

[23] Johansen KL Painter P Delgado C Doyle J. Characterization of physical activity and sitting time among patients on hemodialysis using a new physical activity instrument. J Ren Nutr (2015) 25(1):25–30. doi: 10.1053/j.jrn.2014.06.012 25213326 PMC4282813

[24] Sasaki JE John D Freedson PS. Validation and comparison of ActiGraph activity monitors. J Sci Med Sport (2011) 14(5):411–6. doi: 10.1016/j.jsams.2011.04.003 21616714

[25] Troiano RP Berrigan D Dodd KW Masse LC Tilert T McDowell M. Physical activity in the united states measured by accelerometer. Med Sci Sports Exerc (2008) 40(1):181–8. doi: 10.1249/mss.0b013e31815a51b3 18091006

[26] Task Force of the European Society of Cardiology the North American Society of Pacing Electrophysiology. Heart rate variability. Circulation (1996) 93(5):1043–65. doi: 10.1161/01.CIR.93.5.1043 8598068

[27] Hrubeniuk TJ Sénéchal M Mayo A Bouchard DR. Association between physical function and various patterns of physical activity in older adults: a cross-sectional analysis. Aging Clin Exp Res (2020) 32(6):1017–24. doi: 10.1007/s40520-019-01288-2 31377998

[28] Desrosiers J Bravo G Hébert R Dutil E. Normative data for grip strength of elderly men and women. Am J Occup Ther (1995) 49(7):637–44. doi: 10.5014/ajot.49.7.637 7573334

[29] Manfredini F Mallamaci F D'Arrigo G Baggetta R Bolignano D Torino C. Exercise in patients on dialysis: A multicenter, randomized clinical trial. J Am Soc Nephrol (2017) 28(4):1259–68. doi: 10.1681/ASN.2016030378 PMC5373448 27909047

[30] Vasunilashorn S Coppin AK Patel KV Lauretani F Ferrucci L Bandinelli S. Use of the short physical performance battery score to predict loss of ability to walk 400 meters: Analysis from the InCHIANTI study. Journals Gerontol: Ser A (2009) 64A(2):223–9. doi: 10.1093/gerona/gln022 PMC2655026 19182232

[31] Sheshadri A Kittiskulnam P Johansen KL. Higher physical activity is associated with less fatigue and insomnia among patients on hemodialysis. Kidney Int Rep (2019) 4(2):285–92. doi: 10.1016/j.ekir.2018.10.014 PMC6365400 30775625

[32] Delgado C Johansen KL. Barriers to exercise participation among dialysis patients. Nephrol Dial Transplant (2012) 27(3):1152–7. doi: 10.1093/ndt/gfr404 PMC3289894 21795245

[33] McLlvenny S. Fatigue as a transcultural issue. Eur J Gen Pract (2000) 6(1):20–2. doi: 10.3109/13814780009074502

[34] Tong A Manns B Hemmelgarn B Wheeler DC Evangelidis N Tugwell P. Establishing core outcome domains in hemodialysis: Report of the standardized outcomes in nephrology-hemodialysis (SONG-HD) consensus workshop. Am J Kidney Dis (2017) 69(1):97–107. doi: 10.1053/j.ajkd.2016.05.022 27497527 PMC5369351

[35] Urquhart-Secord R Craig JC Hemmelgarn B Tam-Tham H Manns B Howell M. Patient and caregiver priorities for outcomes in hemodialysis: An international nominal group technique study. Am J Kidney Dis (2016) 68(3):444–54. doi: 10.1053/j.ajkd.2016.02.037 26968042

[36] Fried LP Tangen CM Walston J Newman AB Hirsch C Gottdiener J. Frailty in older adults: Evidence for a phenotype. Journals Gerontol: Ser A (2001) 56(3):M146–M57. doi: 10.1093/gerona/56.3.M146 11253156

[37] Hladek MD Zhu J Crews DC McAdams-DeMarco MA Buta B Varadhan R. Physical resilience phenotype trajectories in incident hemodialysis: Characterization and mortality risk assessment. Kidney Int Rep (2022) 7(9):2006–15. doi: 10.1016/j.ekir.2022.06.009 PMC9459128 36090502

[38] Ramer SJ Scherer JS. Moving the science of patient-reported outcome measures forward: Measuring fatigue in hemodialysis patients. Clin J Am Soc Nephrol (2020) 15(11):1546–8. doi: 10.2215/CJN.14900920 PMC7646242 33174861

